# Effects of different metabolic equivalent of task on glycaemic control in children and adolescents with type 1 diabetes mellitus: a systematic review and meta-analysis

**DOI:** 10.3389/fnut.2026.1963711

**Published:** 2026-10-28

**Authors:** Nianxin Kong, Mingyi Liu, Haipei Gong, Zikang Yin, Jingjie Cai

**Affiliations:** China Youth Sports and the Integration of Sports and Education Public Policy Research Center, Wuhan Sports University, Wuhan, China

**Keywords:** children and adolescents, glycaemic management, HbA1c, metabolic equivalent of task, type 1 diabetes mellitus

## Abstract

**Objective:**

This systematic review and meta-analysis investigated the impact of different metabolic equivalent of task (MET) levels on glycaemic control in children and adolescents with type 1 diabetes mellitus (T1DM).

**Methods:**

Following PRISMA 2020 guidelines, PubMed, Embase, Web of Science, SPORTDiscus, and the Cochrane Library were searched up to July 2025 for randomized controlled trials comparing exercise interventions with routine care in T1DM patients aged 5–18 years. The primary outcome was HbA1c; secondary outcomes included fasting blood glucose, daily insulin dose, and time in range. MET values were calculated as intensity (MET) × duration (minutes) × frequency (per week). Subgroup analyses examined low (<600), moderate (600–1,200), and high (>1,200 METs-min/week) MET levels. Effect sizes were pooled using random-effects models.

**Results:**

Fourteen RCTs involving 543 participants were included. Exercise significantly reduced HbA1c (WMD = −0.83%, 95% CI: −1.34% to −0.33%, *p* < 0.001) and fasting blood glucose (WMD = −41.38 mg/dL, 95% CI: −61.23 to −21.53, *p* < 0.001). Subgroup analyses showed the largest numerical HbA1c reduction in the moderate MET group (WMD = −1.21%, 95% CI: −1.94 to −0.49, *p* < 0.05), followed by the low MET group (WMD = −0.50%, 95% CI: −1.35 to 0.34, *p* > 0.05) and the high MET group (WMD = −0.30%, 95% CI: −1.12 to 0.52, *p* > 0.05); however, the test for differences between subgroups was not statistically significant (*Q* = 3.05 *p* = 0.22).

**Conclusion:**

The moderate MET group showed the largest numerical HbA1c reduction, although between-subgroup differences were not statistically significant. These findings support evidence-based exercise prescription in this population, though further research is needed to clarify dose–response relationships and long-term outcomes.

**Systematic Review Registration:**

Unique Identifier, CRD42024563752.

## Introduction

Type 1 diabetes mellitus (T1DM) is one of the most common chronic autoimmune diseases in children and adolescents, with a global incidence increasing by 3–4% annually. Glycaemic control is a key issue that urgently needs to be addressed in reducing the incidence of complications, disease burden, and long-term health problems in children and adolescents with T1DM. At present, for glycaemic management of children and adolescents with T1DM, insulin therapy, dietary control, and glycaemic monitoring are generally recommended clinically to control glycaemic levels. However, from a long-term management perspective, the compliance with these treatment methods is low. The exercise, as a non-pharmacological intervention that is low-cost and easy to implement, has been widely applied in clinical practice and deeply explored theoretically, and has been verified in numerous studies. Furthermore, the International Society for Pediatric and Adolescent Diabetes (ISPAD) clinical guidelines 2022 clearly indicate that exercise is a key approach for the prevention and control of cardiometabolic risks in children and adolescents with T1DM. Currently, exercise intervention has been recognized as a key approach in the management of adults with T1DM by the American Diabetes Association and the European Association for the Study of Diabetes. It should be particularly noted that, unlike type 2 diabetes mellitus (T2DM), where exercise serves as an initial management strategy, in T1DM, due to absolute insulin deficiency, exercise is primarily an adjunct to insulin therapy rather than an independent treatment, making the definition of its dosage particularly critical.

Multiple systematic reviews and meta-analyses have confirmed the significant effects of exercise intervention on indicators such as HbA1c in T1DM. For instance, García-Hermoso et al. revealed that structured exercise could significantly reduce HbA1c, especially when it was the combined training of aerobic and resistance exercises, high-intensity exercise, and interventions lasting ≥24 weeks (1). Meanwhile, most studies consistently indicated the non-glycaemic benefits of exercise. For instance, moderate-to-vigorous intensity exercise can enhance insulin sensitivity and cut down on the daily insulin dosage. However, most existing studies used single indicators (i.e., duration, frequency, and intensity) or combined indicators to quantify exercise volume when exploring specific effects on glycaemic control, without thoroughly quantifying the integrated relationship between “intensity-duration-frequency.” Consequently, the core question of “what exercise dose can balance metabolic benefits and safety” remains unanswered. Metabolic equivalent of task (MET) holds the key to addressing this issue. Total energy expenditure can be calculated using the formula “exercise intensity (MET value) × duration (minutes) × frequency (times/week)”. This approach not only standardizes dosage criteria across different exercise types but also precisely identifies both the “effective exercise threshold” and “safety boundary.” This provides a quantitative basis for comparing results across studies and for personalized clinical prescription (2, 3). Notably, although existing reviews have revealed the positive effects of exercise on glycaemic control in T1DM, most have not adopted an integrated MET dosage framework, and few have focused on children and adolescents, a population with more pronounced glycaemic fluctuations and higher exercise risks.

Based on this, the present study aimed to comprehensively assess the effects of different exercise volumes on glycaemic control in children and adolescents with T1DM through a systematic review and meta-analysis, quantifying exercise intervention by total exercise volume (METs-min/week), and systematically examining whether glycaemic benefits differ across MET dosage strata. It also aimed to clarify the effects on key glycaemic indicators (e.g., HbA1c, fasting blood glucose, insulin dosage) and to focus on exploring the dose–response relationship. Additionally, it sought to identify participant characteristics and intervention design factors that may influence intervention effects, providing clear evidence for personalised exercise prescription and improved glycaemic management in clinical practice, as well as directions for future research.

## Research design and methods

### Search strategy and selection criteria

The research protocol of this systematic review and meta-analysis has been registered in PROSPERO. This study is strictly conducted in accordance with the revised Preferred Reporting Items for Systematic Reviews and Meta-Analyses (PRISMA) to ensure the transparency and standardization of the research findings.

Five English databases (PubMed, Embase, WOS Core, Sport Discuss, and Cochrane Library) were systematically searched up to July 30, 2025. We searched combined Medical Subject Heading terms and free-text words, employing the following search combinations: children and adolescents AND diabetes AND exercise. Complete search strings for each database are provided in Figure 1.

**Figure 1:**
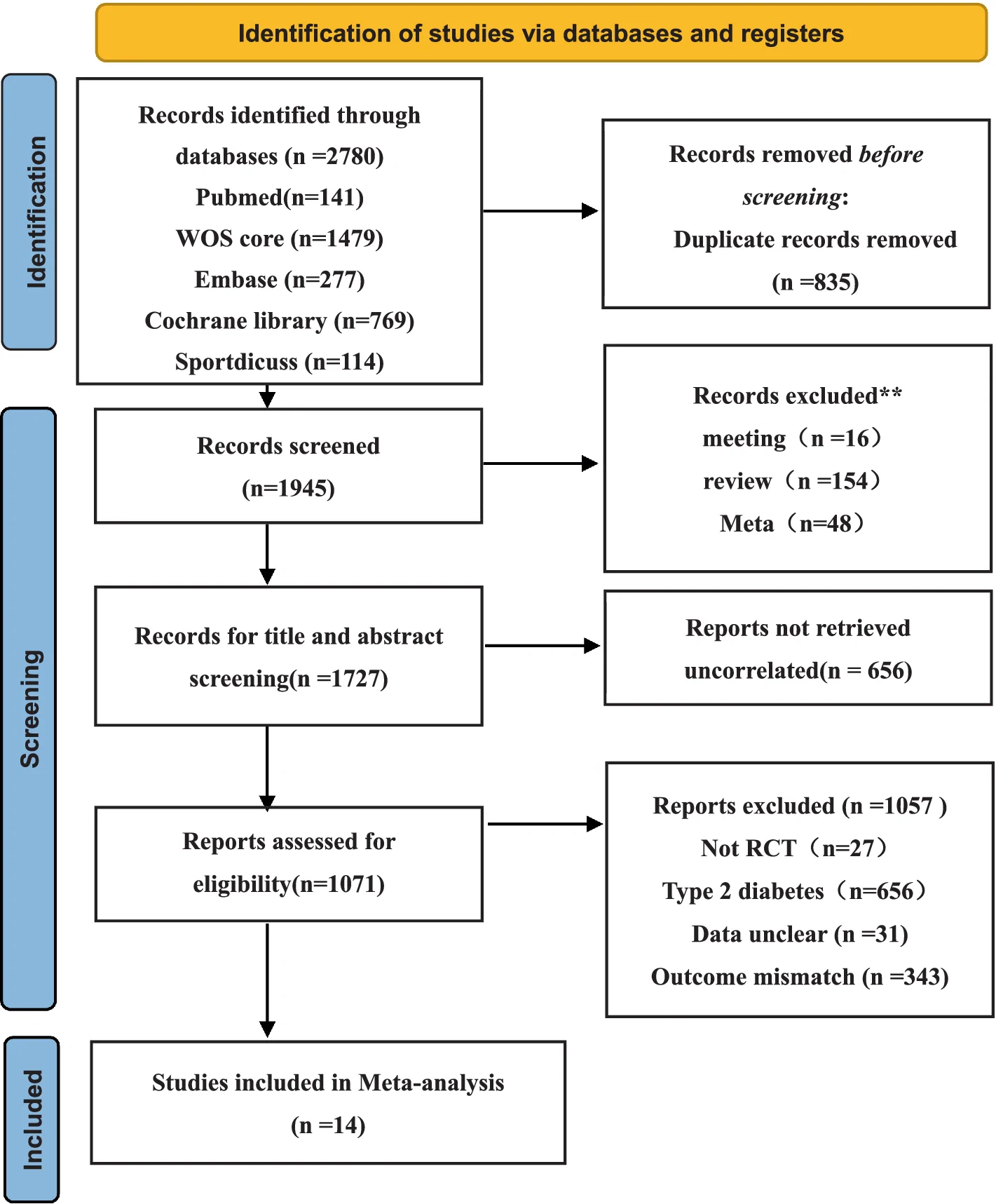
Flow diagram of the included studies.

### Outcome

The inclusion and exclusion criteria were defined using the PICOS principle (Population, Intervention, Comparison, Outcome, and Study design) (Table 1). Studies were included if they were randomized controlled trials (RCTs) that applied different exercise interventions to children and adolescents with T1DM, and reported at least one outcome indicator related to glycaemic control, such as hemoglobin A1c(HbA1c, %), Fasting blood glucose (FBG, mg/dL), daily insulin dose, and time in range (TIR, Unit).

**Table 1:** Inclusion criteria.

Principle	Specific information
Population	Children and adolescents aged 5–18 years with confirmed T1DM were included as study subjects
Interventions	Aerobic exercise, Strength training, or their combination were used as interventions in the study, with an intervention period of at least 1 week
Comparison measures	The control group received usual care, different exercise intervention methods, or other non-exercise intervention measures
Outcome indicators	At least one glycaemic control related indicator was required to be reported in the study, such as HbA1c, Fasting blood glucose (FBG), or insulin sensitivity
Study design	RCTs, studies were limited to published in English

### Data extraction and quality assessment

All identified articles were imported into Note Express version 20.0 to remove duplicates. This research employed an adapted version of the Cochrane Good Practice Data Extraction form, and two researchers independently conducted the data extraction and entry. The information extracted included the content of the pre-designed worksheet, such as publication details, demographic information (i.e., the age of participants, medical conditions, disease duration, etc.), and the details of the intervention and control groups (i.e., intervention method, model, frequency, and duration), as well as the outcome indicators related to glycaemic management (i.e., HbA1c, FBG, daily insulin dose, and TIR). When both parents and children provided reports on glycaemic control outcomes for the latter one, data from parental reports were prioritized for extraction due to their higher reliability. For trials with multiple records, all relevant data were collected and analyzed as part of a single study. If any data were missing, the corresponding authors were contacted via email to request the missing information.

The Cochrane Risk of Bias Tool (ROB 2) was used for RCTs to evaluate five key domains (4): (1) bias from the randomization process; (2) bias from deviations from the intended interventions; (3) bias from missing outcome data; (4) bias in outcome measurement; (5) bias in the selection of reported results. Each of them was categorized as “low risk,” “some concerns” or “high risk.” The overall risk of bias is assigned according to the most severe rating identified in any domain. Specifically, if all domains are rated as low risk, the study will be classified as having an overall low risk of bias. If at least one domain is rated as “some concerns” with no other domains rated as high risk, the study will be classified as having some concerns. If any domain is rated as high risk, the study will be considered to have a high risk of bias. This high risk classification also applies if several domains raise concerns that might significantly affect the study results.

### Data synthesis and analysis

The systematic review and meta-analysis were conducted using statistical software Stata 17.0. MET was an important basis for subgroup analysis in this study. It is a core indicator for quantifying the intensity and total amount of energy expenditure of physical activity, defined relative to the energy expenditure at rest in a sitting position (1 MET = 3.5 mL O₂·kg^−1^·min^−1^). The total MET (unit: METs-min/week) was calculated as the product of exercise intensity (MET), single-session duration (minutes), and weekly frequency (sessions/week). It should be noted that METs-min/week reflects the composite of intensity, duration, and frequency, and should not be interpreted solely as exercise intensity. In this study, with reference to the METs-min stratification criteria of the International Physical Activity Questionnaire (IPAQ) and considering the reduced exercise tolerance characteristic of children and adolescents with T1DM, total exercise volume was divided into three categories for subgroup analysis: low exercise volume (<600 METs-min/week), moderate exercise volume (600–1,200 METs-min/week), and high exercise volume (>1,200 METs-min/week). The IPAQ scoring system defines 600 METs-min/week as the minimum threshold for moderate physical activity, calculated as 4.0 METs×30 min/day×5 days/week = 600 METs-min/week. Because children and adolescents with T1DM generally have lower overall physical activity levels than healthy peers due to increased hypoglycaemia risk and reduced exercise tolerance, the upper limit of the moderate exercise volume category was set at 1200 METs-min/week. (32) conducted a Bayesian dose–response network meta-analysis and showed that in adolescents with T1DM, combined aerobic and resistance training at a dose of 160–1,200 METs-min/week (25–220 min/week) improved glycaemic outcomes while reducing exercise burden. García-Hermoso et al. (1) also confirmed in their meta-analysis that high-intensity exercise, sessions ≥60 min, and interventions lasting ≥24 weeks had more pronounced effects on HbA1c improvement in adolescents with T1DM. Accordingly, these exercise dose categories were defined to provide a quantitative basis for accurately evaluating the effects of different exercise doses on glycaemic control outcomes.

Heterogeneity analysis of the included studies was conducted via Cochran’s *Q* test. The test level was set at *α* = 0.1, and *I*^2^ was used to quantitatively evaluate heterogeneity. If *I*^2^ < 50% and *p* > 0.1, low heterogeneity was considered acceptable, and a fixed effects model (FEM) was used. If *I*^2^ ≥ 50% and *p* < 0.1, high heterogeneity was present, and a random effects model (REM) was selected. The outcome indicators in this study are continuous variables. For effect analysis, the weighted mean difference (WMD) with its 95% confidence interval (CI) was chosen as the effect measure when the same measurement tool was used. When different measurement tools were applied, the standardized mean difference (SMD) and its 95% CI were selected instead. Effect sizes were categorized as follows: a large effect was defined as an SMD or WMD ≥ 0.8; a moderate effect as 0.5 ≤ SMD or WMD < 0.8; a small effect as 0.2 ≤ SMD or WMD < 0.5; and a minimal effect as an SMD or WMD < 0.2. If a trial compared ≥2 items of exercise intervention with one control group, it was regarded as 2 + N independent trials, and the number of participants in the control group was evenly distributed among the trials to avoid double-counting. The robustness of the combined effect size was explored using the sequential elimination method, and the publication bias was tested using the Egger test and funnel plot test. These magnitude descriptors (0.2/0.5/0.8) are conventionally defined for the standardized mean difference (SMD); when applied to the unit-bearing WMD they are used here only as descriptive anchors rather than as thresholds of statistical or clinical significance. For outcomes exhibiting high heterogeneity (*I*^2^ ≥ 50%), we pre-specified subgroup analyses stratified by MET category, intervention type, and participant age, to explore potential sources of heterogeneity.

Because the analysis was performed at the comparison level, the number of comparisons can exceed the number of included studies; for the primary outcome (HbA1c) this yielded 15 comparisons derived from 13 studies (see Primary outcome).

## Results

### Studies characteristics

A total of 14 RCTs focusing on children and adolescents with T1DM were included in this article. These studies were published between 1984 and 2025, involving a total of 543 participants, comprising 254 males (46.8%) and 289 females (53.2%). Specifically, three studies enrolled only male participants, one study enrolled only female participants, and the remaining 10 studies included all genders. The average baseline age of participants was 12.8 ± 2.5 years, ranging from 5 to 18 years. The average duration of T1DM was 5.1 ± 2.4 years. All participants had a disease duration of at least 6 months to ensure a stable baseline condition, with an average baseline HbA1c level of 8.2 ± 1.3% (Table 2).

**Table 2:** Study characteristics.

Source	Country	Sample	Age	Exercise	Frequency	Duration	Period	Outcomes
Campaigne et al (19)	The United States	19	5–11	Aerobic and game-based activities	3 times/week	30 min	12 weeks	①②③
Zimmer et al (20)	Germany Austria	18	15.1 ± 1.1	Aerobic and strength combined workout	3 times/week	60 min	4 weeks	①④
Tomar et al (21)	Saudi Arabia	24	14.27 ± 1.80	Aerobic exercise	3 times/week	60 min	12 weeks	①③
Nazari et al (22)	Iran	40	11.11 ± 2.29	Aerobic and resistance training combined	3 times/week	60 min	16 weeks	①②
Salem et al (23)	Egypt	196	14.78 ± 2.31	Aerobic + Resistance + Balance Training	1 times/week(B) 3 times/week(C)	30 min	24 weeks	①③
Tunar et al (24)	Turkey	31	14.2 ± 2.2 (EG) 14.3 ± 1.8 (CG)	Core Strength + Flexibility Training	3 times/week	40 min	12 weeks	①③
Wong et al (25)	Taiwan, China	28	12.34 ± 2.07	Aerobic exercise	3 times/week	20–30 min	12 weeks	①
Stratton et al (26)	the United States	16	15.1 ± 1.2 (EG) 15.5 ± 0.9 (CG)	Aerobic + Leisure Activities	3 times/week	30–45 min	8 weeks	①②③
Huttunen et al (27)	Finland	32	8.2–16.9	Aerobic and game-based activities	1 times/week	60 min	12 weeks	①
Maurya et al (28)	India	46	13.35 ± 2.41	Comprehensive Sports	5 times/week	60 min	12 weeks	①②③
Parent et al (18)	France Canada	12	9.8 ± 1.4	CME: Fixed-power cycling; IHE: High-intensity cycling + passive recovery alternation	2 times/week	25 min	1 week	④
Nazari et al (29)	Iran	20	12.60 ± 1.34 (OE) 12.40 ± 0.84 (NOLTA)	OE: Resistance training; NOLTA: Aerobic and ball sports	3 times/week	30–40 min(OE); 150 min(NOLTA)	8 weeks	①②
Mohammed et al (30)	Saudi Arabia	40	14.5 ± 1.4 (FDG) 17.8 ± 0.42 (FG) 15.6 ± 1.8 (DG) 14.4 ± 2.0 (CG)	Aerobic+Team Ball Sports, featuring recreational soccer played in small-sided games with 3–5 players per team.	2 times/week (FDG; FG)	90	12 weeks	①②③
Petschnig et al (31)	Austria	21	11.00 ± 0.8 (IG) 11.30 ± 0.7 (CG)	Resistance training	2 times/week	50	32 weeks	①

①HbA1c; ②FBG; ③Daily insulin dose; ④Glucose time in range; EG, Experimental group; CG, Control group; OE, Organized exercise; NOLTA, Non-organized leisure time activity; FDG, Football with diet; FG, Football-only; DG, the Diet-only; IG: Intervention group; B, Attended the exercise sessions once/week; C, Attended the exercise sessions 3 times/week; IHE, Intermittent high-intensity aerobic exercise; CME, continuous moderate-intensity exercise.

### Risk of bias assessment

The results showed that the consensus among researchers reached 96% (*κ* = 0.93), indicating a high level of consistency in the evaluation process. Among the 14 included studies, seven studies (50.0%) were rated as low risk of bias, indicating that these studies met high standards in terms of randomization, intervention bias, missing outcome data, outcome measurement, and selection of outcome reporting. In addition, 6 studies (42.9%) were rated as having some concerns, meaning that these studies had potential risk of bias in certain areas; although these issues might not seriously affect the overall conclusions, attention should be paid when interpreting the results. The remaining 1 study (7.1%) was rated as high risk of bias, mainly due to obvious flaws in its randomization process or outcome reporting. To be specific, 4 studies did not clearly report the generation of random sequence or allocation concealment process, so they were rated as “some concerns” in terms of randomization (Figure 2). Although one study was rated as high risk of bias and four as having some concerns in randomization, all 14 studies were retained because they constitute the available body of evidence on exercise dosing and glycaemic control in paediatric T1D; sensitivity analyses indicated that the pooled HbA1c estimate was robust to their inclusion. Across the five ROB 2 domains, the “some concerns” ratings and the single high-risk rating were confined to the randomization-process domain (unclear random-sequence generation or allocation concealment); no study was rated at high risk for outcome measurement, missing data, or selective reporting. All 14 studies were retained as the available evidence base on paediatric T1D exercise dosing, and the pooled HbA1c estimate remained robust in sensitivity analyses excluding any single study (Figures 3, 4).

**Figure 2:**
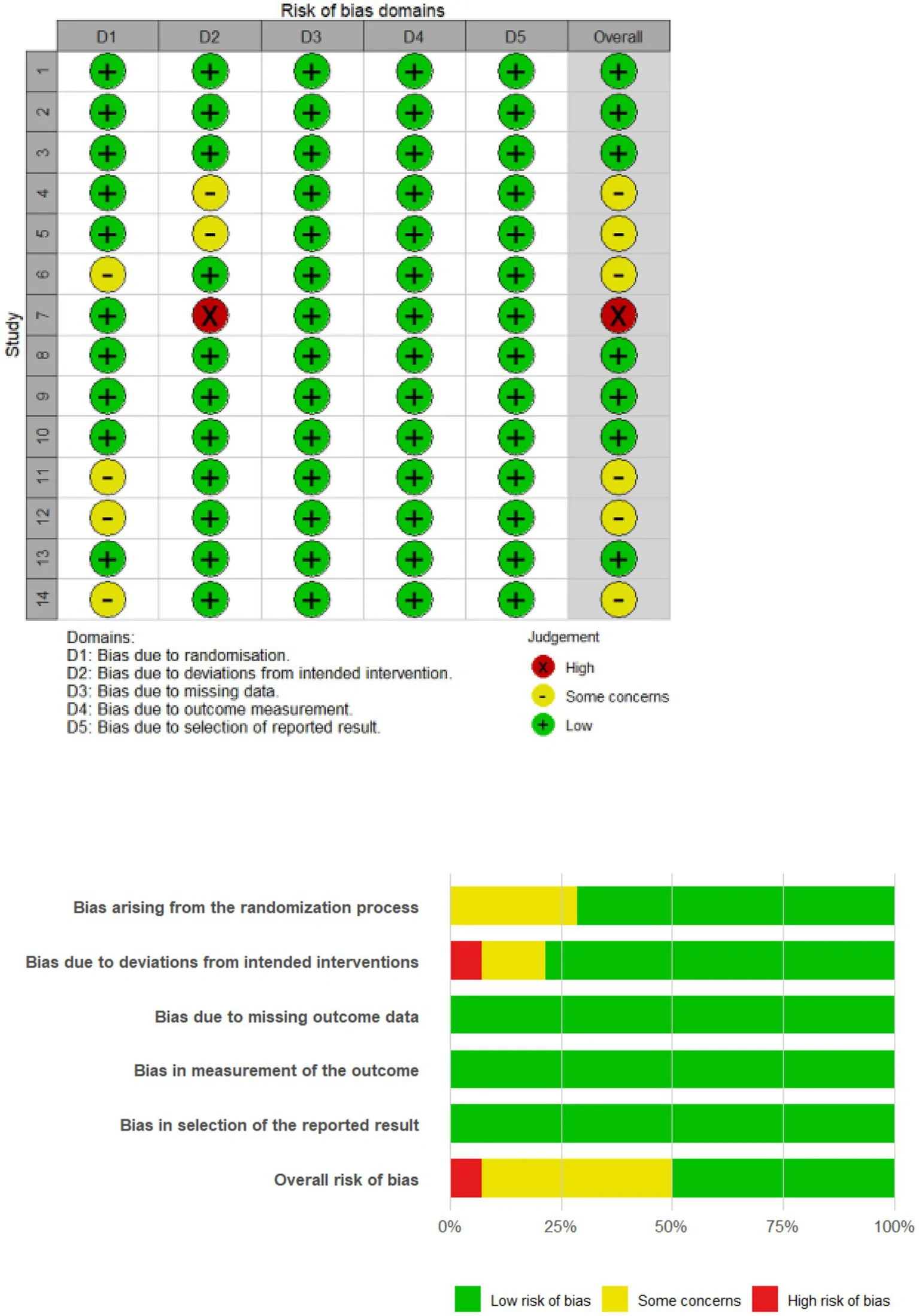
Summary of risk of bias for included studies.

**Figure 3:**
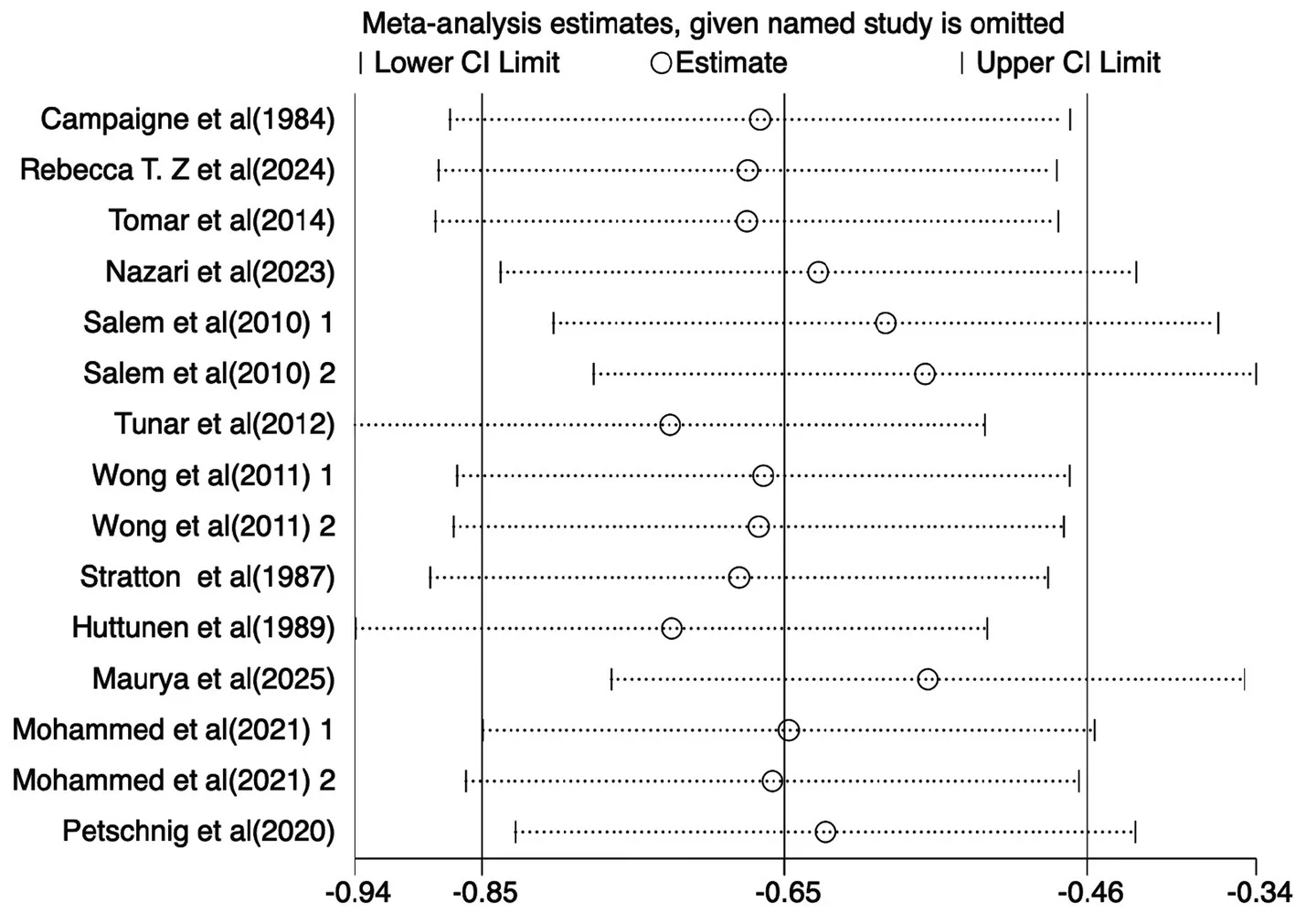
HbA1c sensitivity analysis chart.

**Figure 4:**
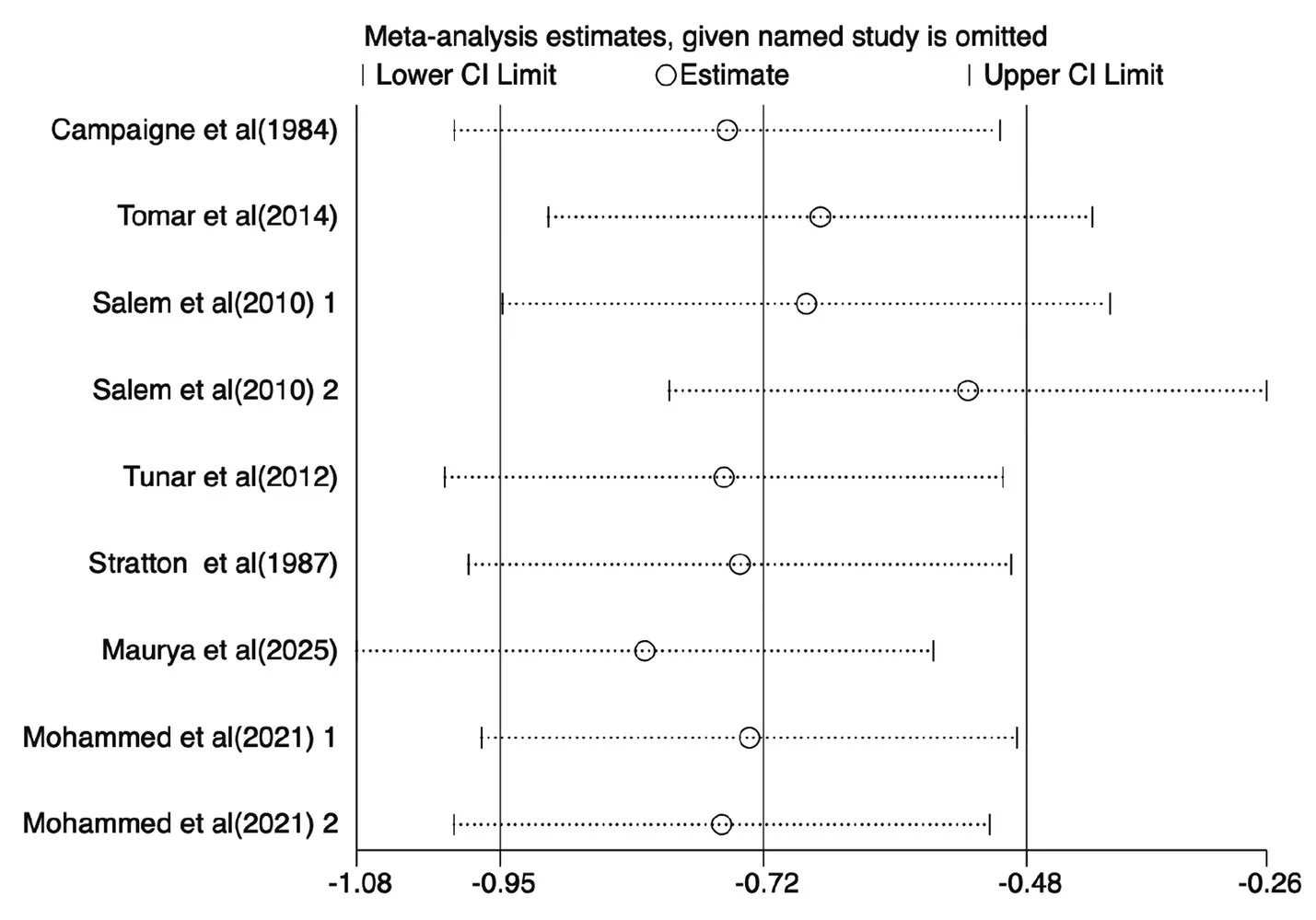
Daily insulin dose sensitivity analysis chart.

### Primary outcome

A total of 15 comparisons from 13 studies involving 543 children and adolescents with T1DM were included to analyze the effect of intervention measures on HbA1c (Figure 5). The results showed that compared with the control group, the HbA1c level in the intervention group was significantly reduced (Weighted Mean Difference = −0.83% 95% CI: −1.34% to −0.33%, *p* < 0.001), indicating that this intervention can effectively improve the long-term glycaemic control level in children and adolescents with T1DM. There was a moderate degree of statistical heterogeneity among the included studies (*I*^2^ = 62.08%, *T*^2^ = 0.52; *Q* = 34.28, df = 14, *p* < 0.001). Referring to the heterogeneity handling methods in similar systematic reviews and meta-analyses, this study adopted a REM for data combination to address this heterogeneity.

**Figure 5:**
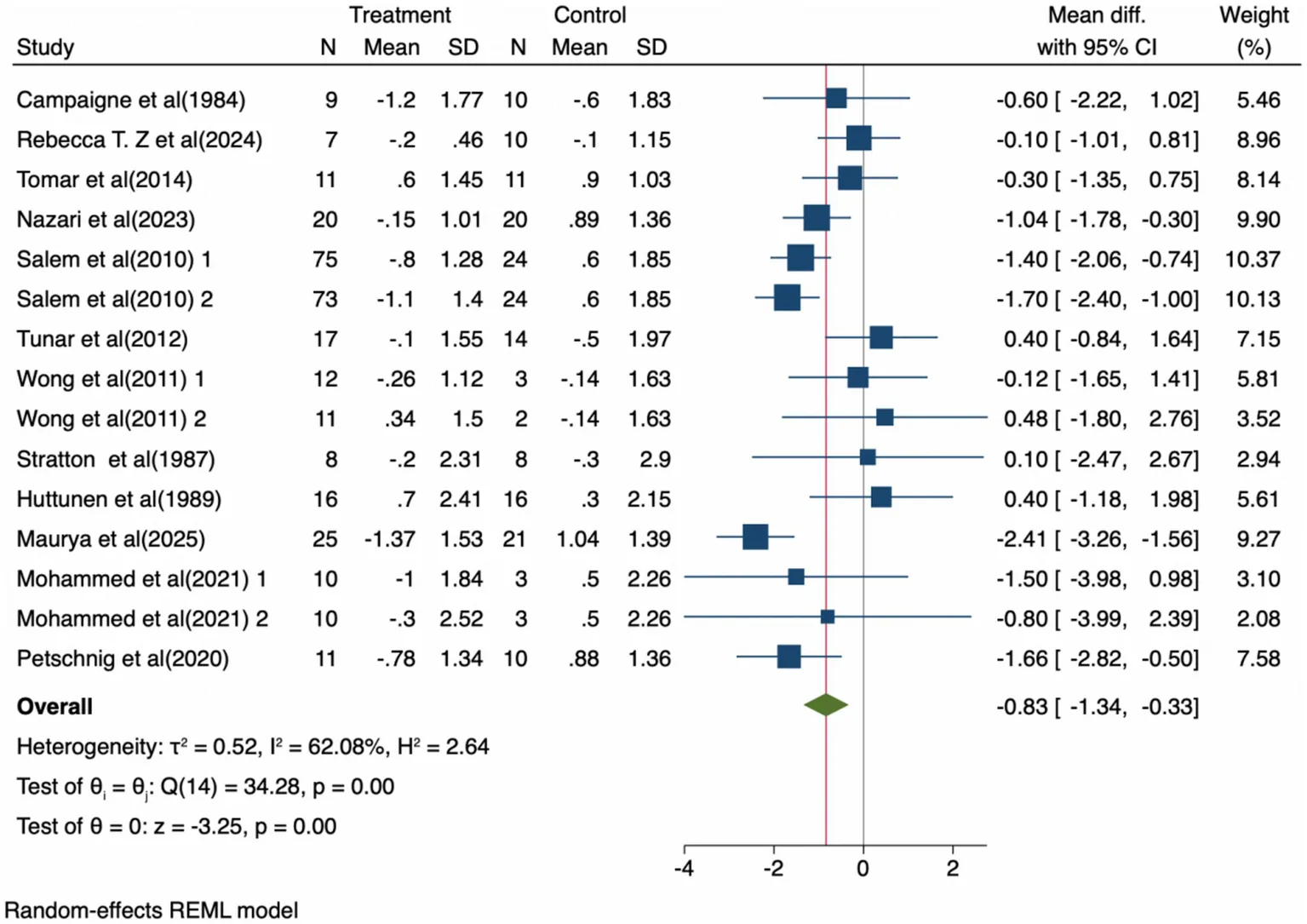
Meta-analysis results comparing HbA1c levels between the exercise intervention group and the control group.

Note on the analytical unit. Throughout this review the meta-analyses were conducted at the comparison level (each exercise intervention arm contrasted with its corresponding control arm) rather than at the study level. Several included RCTs evaluated more than one exercise arm or reported outcomes separately for distinct subgroups, so a single study could contribute more than one comparison; this is why 15 HbA1c comparisons were derived from 13 studies. Moreover, not every one of the 14 studies reported HbA1c as an extractable outcome—the outcomes reported by each study are listed in the Outcomes column of Table 2 (codes ①–④)—and only the HbA1c-reporting studies contributed to these 15 comparisons. Figure 5 presents these 15 arm-level comparisons in a forest plot; its entry count therefore reflects the comparison level and is not expected to equal the 14 study-level entries in Table 2.

### Secondary and safety outcomes

#### Daily insulin dose

A total of 10 comparisons were included and analyzed using the random-effects REML model. The results showed that compared with the control group, the daily insulin dose in the intervention group decreased (Weighted Mean Difference = −0.16 U/kg/d 95% CI: −0.31 to −0.01, *p* = 0.04). Although it did not reach statistical significance, the negative effect size still suggested that exercise intervention had a tendency to reduce insulin dose. The results of the heterogeneity test showed high heterogeneity among the studies (*T*^2^ = 0.03, *I*^2^ = 82.39%, *H*^2^ = 5.68; *Q*(8) = 30.85, *p* < 0.001) (Figure 6).

**Figure 6:**
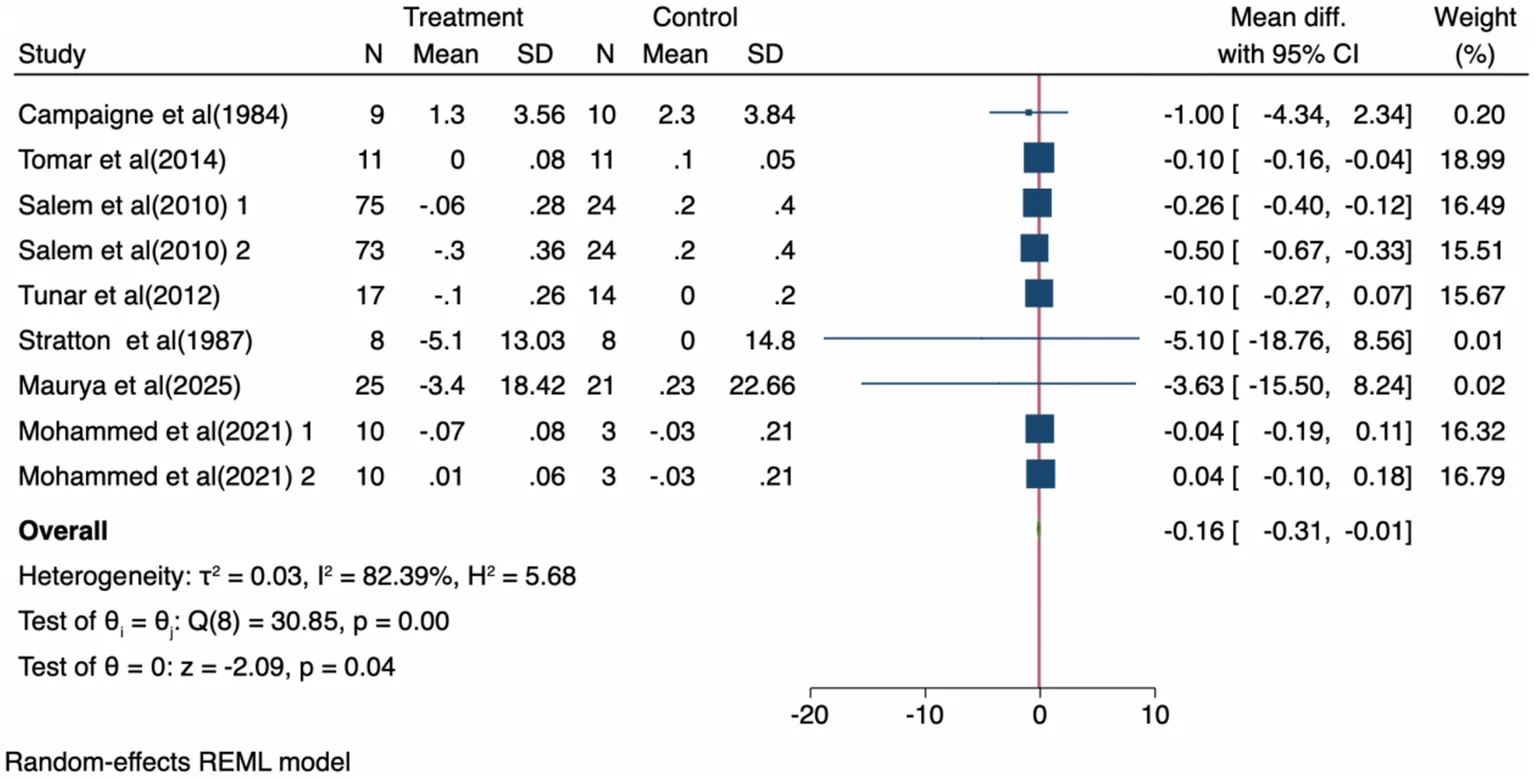
Meta-analysis results comparing insulin doses between the exercise intervention group and the control group.

#### FBG

A total of 6 comparisons were included and analyzed using a fixed-effects inverse variance model. Compared with the control group, the FBG in the intervention group decreased significantly (Weighted Mean Difference = −41.38 mg/dL, 95% CI: −61.23 to −21.53, *p* < 0.001), the difference was statistically significant, and the result was less affected by heterogeneity, providing reliable evidence for exercise-assisted glycaemic control. The test for heterogeneity among studies showed that *I*^2^ = 0.00%, *H*^2^ = 1.00, *Q*(6) = 5.62, and *p* = 0.47, indicating no heterogeneity among studies (Figure 7).

**Figure 7:**
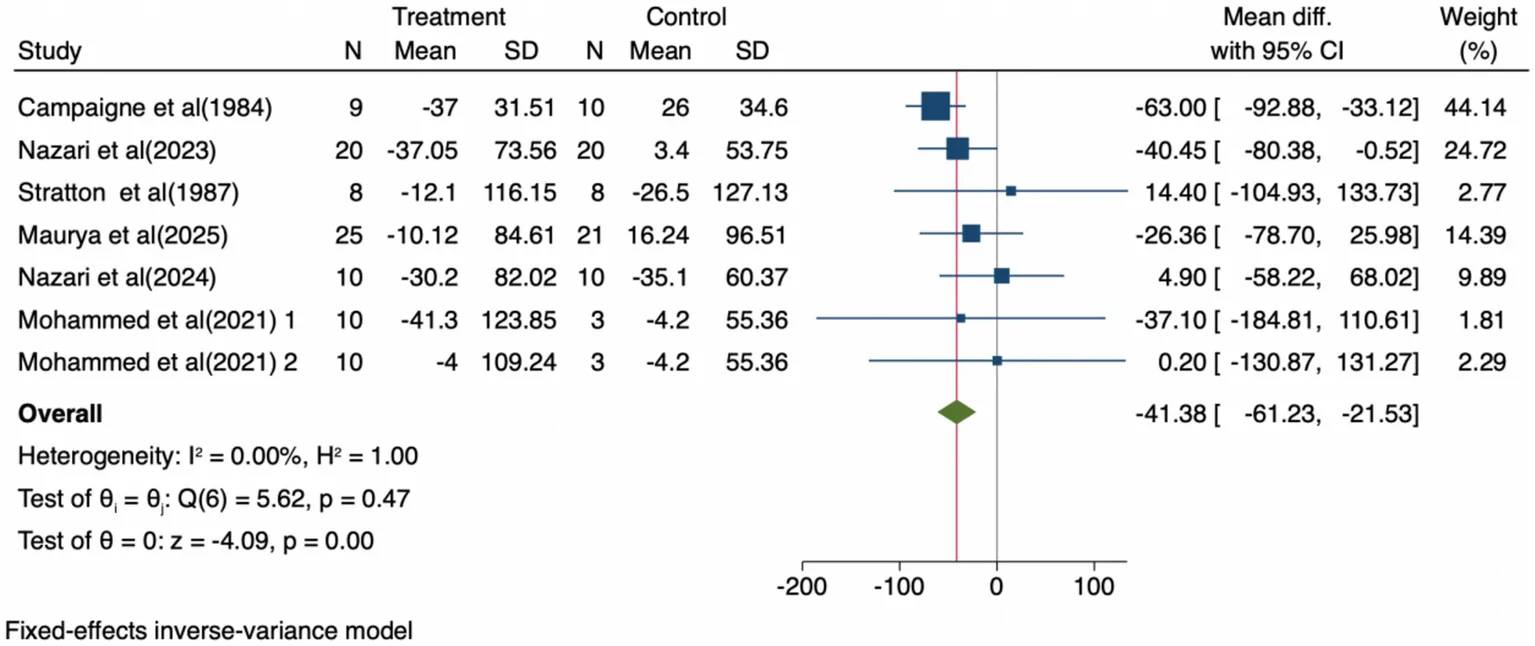
Meta-analysis results comparing FBG levels between the exercise intervention group and the control group.

#### TIR

Three comparisons were included and analyzed using a fixed-effects inverse variance model. The results showed that the intervention group did not exhibit statistically significant differences in glycaemic control within the target time range compared to the control group. (Weighted Mean Difference = 8.23%, 95% CI: −5.12 to 21.59, *p* = 0.23), that is, it cannot be considered that the intervention has a significant impact on this outcome indicator. Heterogeneity analysis among studies revealed *I*^2^ = 30.45%, *H*^2^ = 1.44, *Q*(2) = 2.88, and *p* = 0.24, suggesting a low heterogeneity across studies(Figure 8).

**Figure 8:**
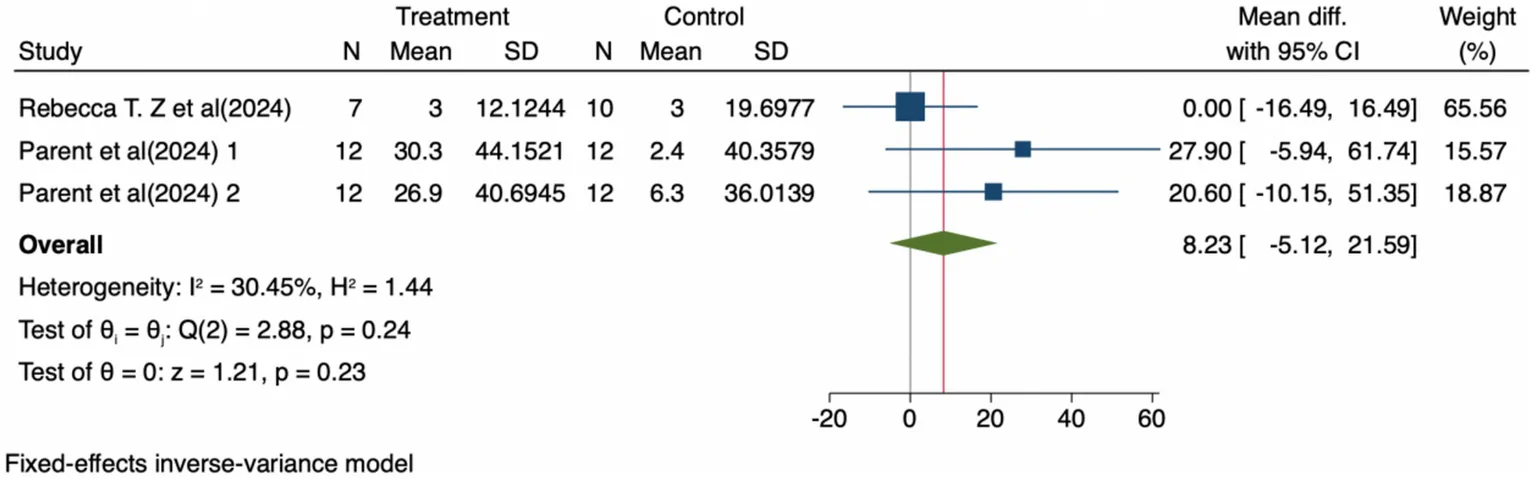
Meta-analysis results comparing time in range (TIR) between the exercise intervention group and the control group.

#### Subgroup analysis

In this study, according to the MET in units of METs-min/week, the included studies were divided into 3 subgroups, namely the low, medium, and high MET subgroups. REM was used to analyze the effect of different MET on HbA1c in children and adolescents with T1DM (Figure 9).

**Figure 9:**
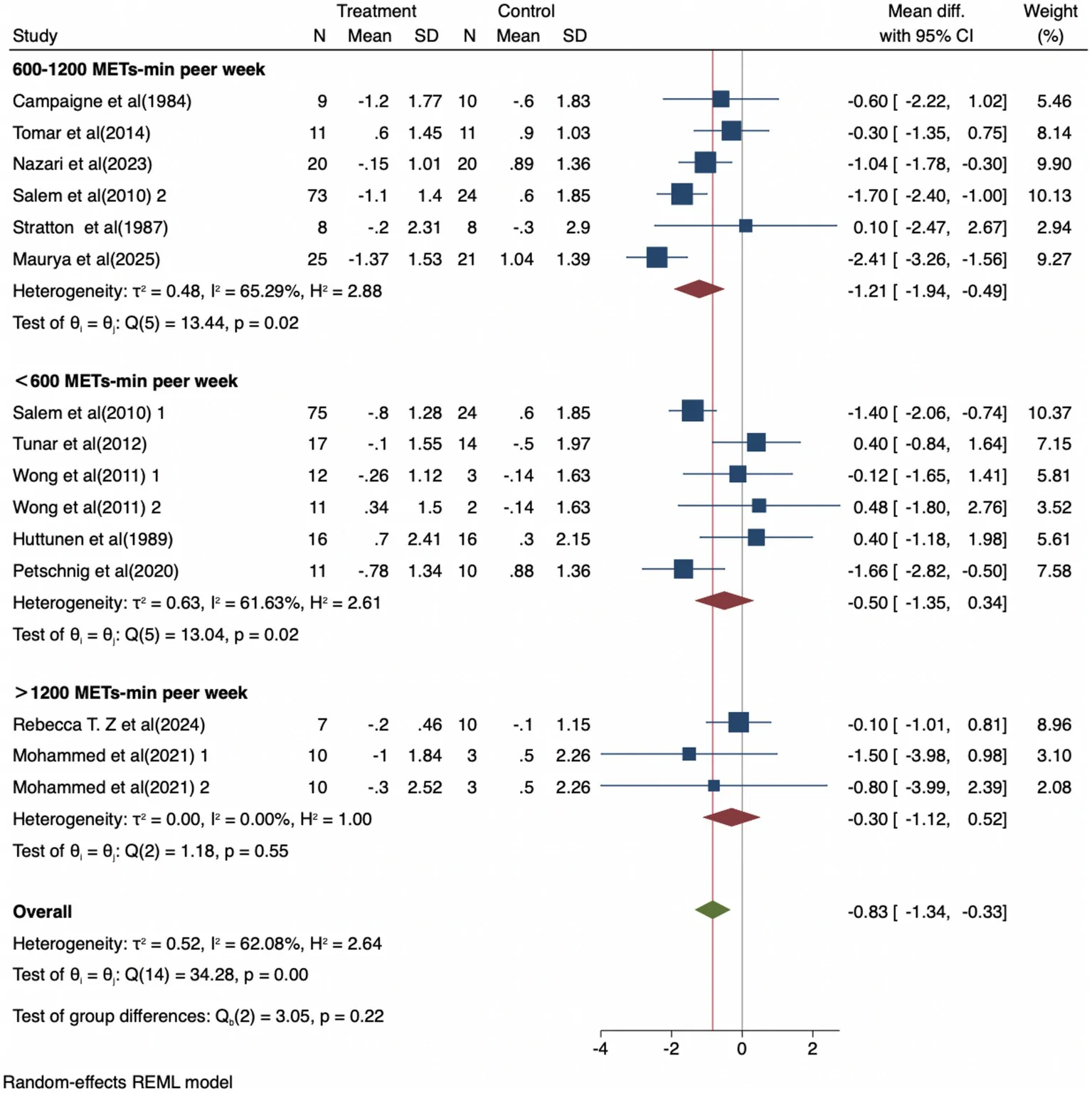
Subgroup analysis results for different physical activity equivalents.

For the moderate MET subgroup, a total of 6 comparisons were included. Results showed that the HbA1c of this subgroup significantly decreased after intervention compared with the control group (Weighted Mean Difference = −1.21%, 95% CI: −1.94 to −0.49, p<0.05). Heterogeneity tests across studies revealed *T*^2^ = 0.48, *I*^2^ = 65.29%, *H*^2^ = 2.88, and *Q*(5) = 13.44 (*p* = 0.02), indicating a moderate heterogeneity, and the interventions within this MET range significantly reduced HbA1c levels. The low exercise dosage subgroup, which included 6 comparisons, showed no significant effect on HbA1c (Weighted Mean Difference = −0.50%, 95% CI: −1.35 to 0.34, *p* > 0.05). Heterogeneity tests across studies revealed *T*^2^ = 0.63, *I*^2^ = 61.63%, *H*^2^ = 2.61, *Q*(5) = 13.04 (*p* = 0.02), indicating a moderate heterogeneity. The high MET subgroup included three comparisons, with results indicating that interventions within this range had no significant effect on HbA1c (Weighted Mean Difference = −0.30%, 95% CI: −1.12 to 0.52, *p* > 0.05). Heterogeneity tests showed *T*^2^ = 0.00, *I*^2^ = 0.00%, *H*^2^ = 1.00, *Q*(2) = 1.18 (*p* = 0.55), indicating no heterogeneity among studies. Tests for differences between groups showed *Q_b_*(2) = 3.05, *p* = 0.22, suggesting that the effect differences between different MET subgroups were not statistically significant. Overall, moderate MET may have a numerically greater reduction in HbA1c, but due to the lack of statistical significance in differences between subgroups, more studies are needed to further verify the specific effects of different MET.

The three MET subgroups were defined for every study by its total MET (METs-min/week) as low (<600), moderate (600–1,200), or high (>1,200), with the per-study classification detailed in the MET classification Table 3.

**Table 3:** Classification of included studies by exercise dose (METs-min/week = MET × duration × weekly frequency).

Source	Exercise protocol	MET	METs-min/Week	MET category
Campaigne et al. (19)	3 sessions/week, 30 min/session, vigorous	7 (high, >6 METs)	7 × 30 × 3 = 630	Moderate (600–1,200)
Zimmer et al. (20)	3 sessions/week, 60 min/session, track training (high)	8 (high)	8 × 60 × 3 = 1,440	High (>1,200)
Tomar et al. (21)	3 sessions/week, 60 min/session, low–moderate (40–60% HRmax)	4 (moderate, 3–6 METs)	4 × 60 × 3 = 720	Moderate (600–1,200)
Nazari et al. (22)	3 sessions/week, 60 min/session, resistance + aerobic (moderate)	5 (moderate)	5 × 60 × 3 = 900	Moderate (600–1,200)
Salem et al. (23) (group B)	1 session/week, ~60 min/session (moderate)	6 (moderate–high)	6 × 60 × 1 = 360	Low (<600)
Salem et al. (23) (group C)	3 sessions/week, ~60 min/session (moderate)	6 (moderate–high)	6 × 60 × 3 = 1,080	Moderate (600–1,200)
Tunar et al. (24)	3 sessions/week, 45 min/session, Pilates (low–moderate)	3 (low, 2–3 METs)	3 × 45 × 3 = 405	Low (<600)
Wong et al. (25)	3 sessions/week, 20–30 min/session (moderate, taken as 30 min)	4 (moderate)	4 × 30 × 3 = 360	Low (<600)
Stratton et al. (26)	3 sessions/week, 30–45 min/session (moderate, taken as 45 min)	5 (moderate)	5 × 45 × 3 = 675	Moderate (600–1,200)
Huttunen et al. (27)	1 session/week, 60 min/session (moderate)	4 (moderate)	4 × 60 × 1 = 240	Low (<600)
Maurya et al. (28)	5 sessions/week, 60 min/session, yoga (low)	2 (moderate)	2 × 60 × 5 = 600	Moderate (600–1,200)
Nazari et al. (29)	3 sessions/week, body-weight resistance (aerobics), 30 min	4 (moderate)	4 × 30 × 3 = 360	Low (<600)
Mohammed et al. (30)	2 sessions/week, small-sided games (high-intensity interval), 90 min	8 (high)	8 × 90 × 2 = 1,440	High (>1,200)
Petschnig et al. (31)	2 sessions/week, machine strength training (moderate), 50 min	4 (moderate)	4 × 50 × 2 = 400	Low (<600)

### Sensitivity analyses and publication bias

Stata 17 was used for sensitivity analysis and indicated that the meta-results were rather stable; while any literature was excluded, the overall results did not change significantly (Figures 3, 4).

### Publication bias

The funnel plot results indicated that the effect size distribution of the included studies did not fully form a symmetrical “funnel” shape. To be specific, studies with medium sample sizes and significant effect sizes clustered in the upper-middle area of the funnel, while small-sample studies were relatively scattered. Some study points that might represent negative results or small effect sizes were absent from the funnel plot, suggesting a potential risk of publication bias. The sources of bias may include: small-sample studies showing insignificant effects of exercise interventions on glycaemic control in children and adolescents with T1DM, short follow-up periods leading to minimal changes in outcome measures, or research design flaws (Figure 10).

**Figure 10:**
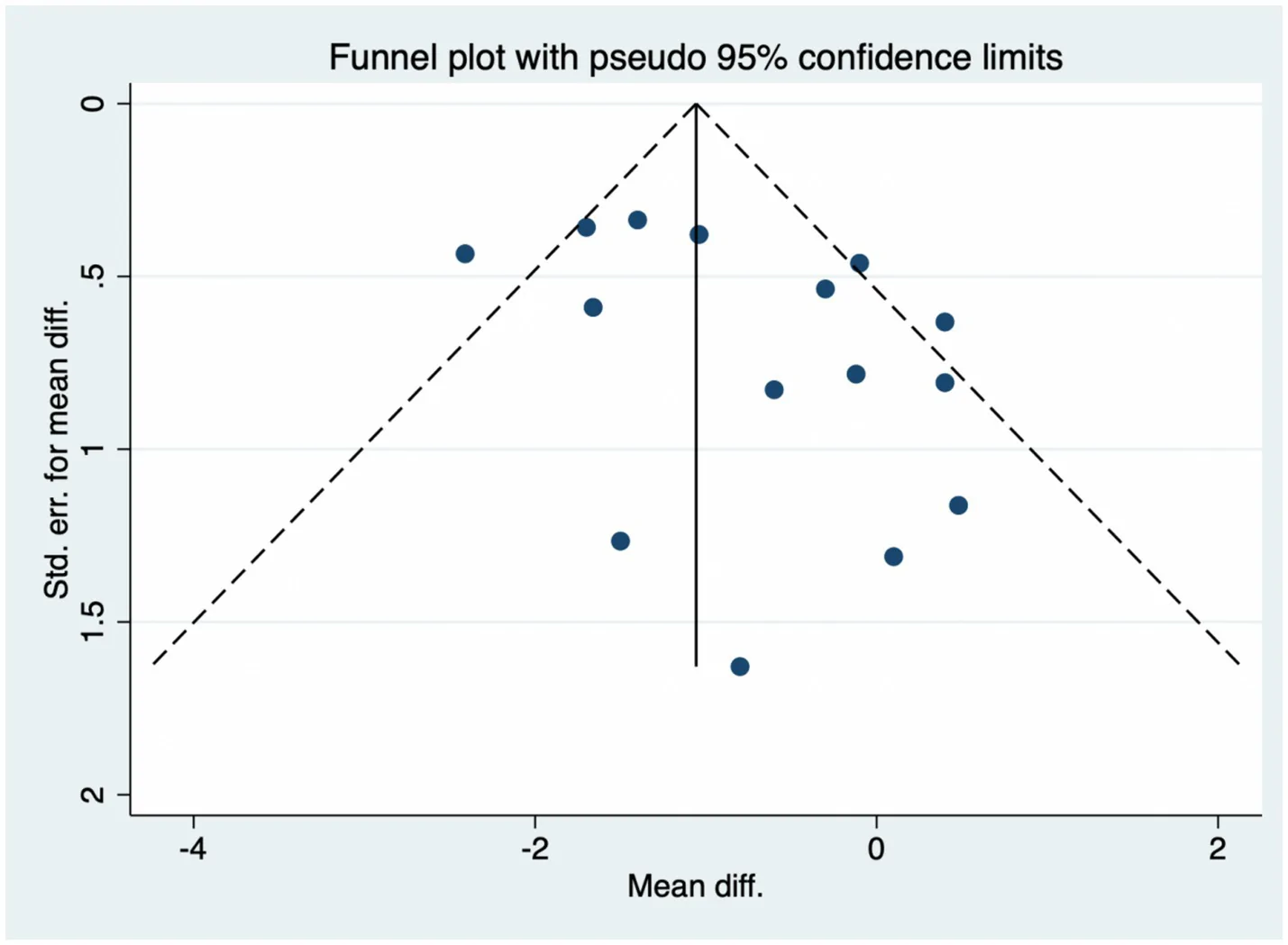
HbA1c funnel plot.

## Discussion

This systematic review and meta-analysis focuses on the impact of different MET on glycaemic management in children and adolescents with T1DM. It comprehensively integrates existing research evidence, providing important evidence-based support for the clinical practice of exercise intervention in the population, and supplements the deficiencies in the field research in terms of the subdivision of MET and the joint analysis of multiple outcome indicators. It should be emphasized that the HbA1c reduction in children and adolescents with T1DM is influenced by multiple factors—including diet, diabetes education, genetic background, and ethnicity—and cannot be attributed solely to exercise equivalent (MET). Therefore, the relationship between different MET levels and glycaemic outcomes should be interpreted in conjunction with these factors.

Regarding the impact of exercise interventions on outcome indicators, HbA1c serves as a key indicator for assessing long-term glycaemic control in people with diabetes. The sustained effect of observational exercise intervention on glycaemic control in children and adolescents with T1DM is significant (5). The results of this study show that exercise significantly improves HbA1c levels, which aligns with findings from meta-analyses on exercise intervention in children and adolescents with T1DM conducted by García-Hermoso et al. (1), Quirk et al. (6) that similarly verified that exercise helps lower HbA1c by optimizing the body’s ability to maintain stable glycaemic metabolism. For example, the meta-analysis by García-Hermoso et al. demonstrated that structured exercise training significantly reduces HbA1c levels, with greater improvements observed in regimens combining aerobic and strength training, high-intensity exercise, intervention duration ≥24 weeks, and single sessions ≥60 min.

Quirk’s meta-analysis further confirmed that exercise interventions for children and adolescents with T1DM yield a standardized mean difference of −0.52 (95% CI − 0.97 to −0.07) in HbA1c, an effect that reflects the optimization of glycaemic metabolic homeostasis and thereby provides additional support for the role of exercise in improving HbA1c in this population. Those studies have reached similar findings because exercise effectively activates key signaling pathways in the skeletal muscles of children and adolescents with T1DM. This activation helps GLUT work more efficiently, boosting the muscles’ ability to take in glucose. At the same time, the insulin signaling pathway is optimized, and insulin sensitivity is enhanced, thereby fundamentally reshaping the homeostasis of glucose metabolism, resulting in a significant improvement in the disorder of glucose metabolism in the body, which is highly in line with the metabolic physiological mechanism by which exercise promotes glycaemic management (7, 8). FBG is a key measure for assessing glycaemic levels in the basic resting state (9). It directly shows the balance between liver glycogen breakdown and the body’s baseline insulin secretion (10), making it essential for evaluating how well exercise intervention regulates basic glucose metabolism in children and adolescents with T1DM. After exercise intervention, FBG levels were found to decrease significantly in this study. This result aligns with the findings of MacMillan et al., who also reported that regular exercise effectively lowers FBG levels in adolescents with T1DM (11).

Diet is an important independent factor influencing HbA1c that cannot be overlooked. The systematic review and meta-analysis by Cruz Castillo et al. included eight studies in adults with T1DM and found that a low-carbohydrate diet (LCD, approximately 50–100 g carbohydrates per day) for 12 weeks significantly reduced HbA1c (point estimate 0.79 mmol/mol, 95% CI 0.57–1.02, *p* < 0.001) and total daily insulin dose (point estimate 0.96 U, 95% CI 0.67–1.25, *p* < 0.001), with no significant effects on blood lipids, body weight, or BMI (12). This study indicates that carbohydrate restriction lowers HbA1c by reducing postprandial glycaemic excursions and improving fasting glucose and glycaemic variability, independently of exercise. Consequently, among the children and adolescents with T1DM included in this study, differences across trials in dietary carbohydrate intake, accuracy of carbohydrate counting, insulin-to-carbohydrate ratios, and dietary education may independently affect HbA1c and insulin dose, representing important confounders beyond MET. Notably, ISPAD recommends that carbohydrate intake should account for 40–50% of total energy in paediatric T1DM and discourages excessive carbohydrate restriction that may impair growth and development. Future studies should therefore record dietary intake in detail and control for dietary factors in the analysis.

Diabetes education is likewise a key determinant of HbA1c reduction. Cruz Castillo et al. (12) pointed out that intensive insulin therapy depends on accurate carbohydrate counting and insulin-to-carbohydrate ratios to calculate prandial insulin doses; both basic carbohydrate counting (BCC) and advanced carbohydrate counting (ACC) are associated with lower HbA1c. Structured diabetes education can improve patients’ self-management capabilities, such as glycaemic monitoring before and after exercise, recognition and treatment of hypoglycaemia, and insulin dose adjustment—thereby influencing the adherence to and glycaemic effect of exercise interventions. Differences across trials in the level of diabetes education, educational model, and patients’ self-management ability may therefore partly explain the heterogeneity in HbA1c reduction and cannot be attributed solely to MET.

Genetic background and ethnic differences also significantly affect HbA1c levels and their reduction. The review by Soranzo reported that the heritability of HbA1c reaches 47–59%, higher than that of fasting glucose (34–36%) and 2-h OGTT glucose (33%) (13). Genome-wide association studies have identified multiple loci associated with HbA1c, including GCK, G6PC2, MTNR1B, HK1, HFE, TMPRSS6, ANK1, SPTA1, FN3K, and TCF7L2; some of these act through glycaemic pathways, whereas others act through non-glycaemic pathways such as erythrocyte lifespan, haemoglobin concentration, iron metabolism, and erythrocyte membrane stability. For example, HK1, HFE, and TMPRSS6 influence HbA1c independently of glucose. Although African Americans have higher HbA1c levels than Europeans, genetic ancestry explains only about 1% of the variation, with socio-economic, demographic, and metabolic factors playing larger roles. Nevertheless, genetic variation may still affect individual HbA1c differences and diabetes diagnostic classification (approximately 2%). The HbA1c reduction in children and adolescents with T1DM of different ethnicities may therefore be influenced by genetic background, and the effect of exercise intervention cannot be attributed solely to MET. Future studies should incorporate ethnicity, genetic markers, and non-glycaemic factors and conduct stratified analyses.

The consistent results stem from exercise’s ability to strengthen insulin’s suppression of hepatic glycogen release, boost basal glucose uptake in peripheral tissues, and alleviate fasting glucose metabolism disorders. This mechanism has been widely confirmed in diabetes exercise metabolism research (14). Both our study and earlier work have demonstrated exercise’s regulatory impact on FBG, supporting the general applicability of this metabolic pathway. Insulin dose serves as a direct measure of exogenous insulin dependence in glycaemic management for children and adolescents with T1DM. By tracking daily insulin use, it effectively shows how Exercise intervention improves the body’s natural glycaemic control abilities (15). In T1DM, inadequate insulin production and insulin resistance occur together. Exercise helps reduce the need for extra insulin by making cells more sensitive to insulin and increasing glucose use. For this reason, Insulin dose is a key indicator for assessing the metabolic benefits of Exercise intervention.

A statistically significant reduction in insulin dose was observed with exercise in this study, and the effect was consistent with the hypothesis that “exercise refines glycaemic management and reduces insulin dependence,” though results were influenced by high heterogeneity across studies. TIR, a new dynamic glycaemic monitoring tool, precisely shows how well glycaemic is controlled and the risk of fluctuations by measuring how much time glucose stays within the ideal 3.9–10.0 mmol/L range (16). This makes it very important for assessing how exercise intervention can improve the precision of glycaemic management in children and adolescents with T1DM. However, in terms of glycaemic TIR, no significant improvement was observed in exercise either, which is inconsistent with the conclusion in some studies that exercise can increase TIR. It is speculated that this is related to the high heterogeneity among the included studies, as differences in TIR measurement methods, exercise intervention details, and other aspects of different studies have masked the potential positive effect of exercise.

Subgroup analysis results showed that moderate MET showed the largest numerical improvement in the health of children and adolescents with T1DM, low MET leads to only slight improvements, while high MET has no statistically significant effect on HbA1c. Among them, the significant improvement of moderate MET on HbA1c is in line with the view put forward by some studies that “moderate exercise has more favorable glycaemic effects in diabetic patients”, probably because this kind of exercise intensity can better balance the metabolic stimulation of exercise and the body’s tolerance. The core mechanism is that the intervention in the medium MET range can continuously activate the AMPK pathway, promoting the translocation of GLUT4 to the cell membrane, enhancing insulin-mediated glucose uptake and utilization, and effectively improving the long-term glycaemic management of children and adolescents with T1DM. Meanwhile, despite variations in moderate MET due to differences in exercise intensity and intervention frequency, the dose range of “600–1,200 METs-min/week” has been shown to overcome individual differences and trigger a stable improvement in glucose metabolism. This finding aligns closely with classic research on metabolic regulation pathways in the field of exercise and diabetes management. For low MET, previous studies have explored their intervention effects on HbA1c in children and adolescents with T1DM. For instance, MacMillan et al. reviewed a low-intensity exercise intervention among adolescents with T1DM, aiming to verify whether basic exercise stimulation could improve glycaemic management.

We found in this study that the improvement effect of low MET on HbA1c was weak and not statistically significant, and was significantly affected by the heterogeneity among studies. This finding was in line with the conclusion of MacMillan et al. (11) that both of them found that the improvement effect of low MET on HbA1c was unstable. The core reason lies in that the low MET fails to provide continuous and sufficient metabolic stimulation for the body, and it is difficult to break through the pathological state of glucose metabolism disorders in children and adolescents with T1DM, such as insufficient insulin secretion and peripheral tissue resistance. Specifically, the improvement effect on HbA1c is not significant because low MET fails to effectively activate the glucose uptake pathway in skeletal muscle and cannot significantly enhance insulin sensitivity. Meanwhile, differences in exercise types and intervention durations among studies further increase heterogeneity and make the effect more unstable. For high MET, existing studies have looked into its potential to improve HbA1c in children and adolescents with T1DM, suggesting that “higher intensity exercise may help break the bottleneck in glycaemic management”. However, some other studies have found that the effect of high exercise capacity is controversial.

To be specific, the moderate-MET subgroup achieved a statistically significant HbA1c reduction, whereas the high-MET subgroup did not. The significant moderate-intensity effect is consistent with the broader evidence that physical activity is generally associated with improved glycaemic control in youth with T1DM Huerta-Uribe et al. (17), whereas the null high-MET finding indicates that the dose–response relationship plateaus or remains uncertain at higher exercise volumes. This result should be interpreted with caution for two reasons. First, the high-MET subgroup comprised only 3 comparisons and was therefore underpowered, with a wide confidence interval, so non-significance does not necessarily imply the absence of a benefit. Second, exercise intensity and dose are known to markedly shape acute glycaemic responses in youth with T1DM Parent et al. (18); high-intensity exercise can elicit acute glycaemic variability—for example, transient catecholamine-driven hyperglycaemia and a subsequent risk of hypoglycaemia—which may complicate day-to-day glycaemic management and offset the net long-term benefit on HbA1c. Larger, well-powered trials are therefore warranted to clarify the effect of high exercise doses on glycaemic control in children and adolescents with T1DM.

The study has several limitations. The limited number of included studies and the small sample size of some subgroup studies may affect the stability of the results; the heterogeneity between the implementation details of each study’s exercise intervention and the baseline characteristics of the research subjects also increases the difficulty in interpreting the results. Future research should involve larger sample sizes and include multi-center, large-scale prospective studies. It should also improve study designs with stricter standardization of exercise intervention, and further investigate the physiological mechanisms through which different MET impact various outcome indicators. Meanwhile, long-term follow-up studies are needed to determine the long-term effects and potential adverse reactions of exercise. These steps will help better guide the development of clinical exercise intervention programs and support glycaemic management in children and adolescents with T1DM.

## Conclusion

Our study demonstrates that exercise can effectively improve glycaemic control in children and adolescents with T1DM, significantly reducing both HbA1c and FBG levels. The reduction in daily insulin dosage reached statistical significance, while no significant impact on time spent within the target glycaemic range was noted. Further high-quality studies with more standardized intervention details are needed to clarify the comprehensive role of exercise in glycaemic management for T1DM children and adolescents. Subgroup analysis by MET revealed that the moderate group significantly reduced HbA1c. This finding provides evidence-based support for quantifying clinical exercise prescriptions. More high-quality studies will be needed to validate its long-term value and advance precision-based exercise interventions for children and adolescents with T1DM.

## Data Availability

The original contributions presented in the study are included in the article/supplementary material, further inquiries can be directed to the corresponding author/s.

## References

[1] García-Hermoso A Ezzatvar Y Huerta-Uribe N Alonso-Martínez AM Chueca-Guindulain MJ Berrade-Zubiri S . Effects of exercise training on glycaemic control in youths with type 1 diabetes: a systematic review and meta-analysis of randomised controlled trials. Eur J Sport Sci. (2023) 23:1056–67. doi: 10.1080/17461391.2022.2086489, 35659492

[2] Ainsworth BE Haskell WL Herrmann SD Meckes N Bassett DR Jr. Tudor-Locke C . Compendium of Physical Activities: a second update of codes and MET values. Med Sci Sports Exerc. (2011) 43:1575–1581. doi: 10.1249/MSS.0b013e31821ece12, 21681120

[3] Wasfy MM Baggish AL. Exercise dose in clinical practice. Circulation. (2016) 133:2297–313. doi: 10.1161/CIRCULATIONAHA.116.018093, 27267537 PMC4902280

[4] Sterne JAC Savović J Page MJ Elbers RG Blencowe NS Boutron I . RoB 2: a revised tool for assessing risk of bias in randomised trials. BMJ. (2019) 366:l4898. doi: 10.1136/bmj.l4898, 31462531

[5] Czenczek-Lewandowska E Grzegorczyk J Mazur A. Physical activity in children and adolescents with type 1 diabetes and contemporary methods of its assessment. Pediatr Endocrinol Diabetes Metab. (2018) 24:179–84. doi: 10.5114/pedm.2018.83364, 30963755

[6] Quirk H Blake H Tennyson R Randell TL Glazebrook C. Physical activity interventions in children and young people with type 1 diabetes mellitus: a systematic review with meta-analysis. Diabet Med. (2014) 31:1163–73. doi: 10.1111/dme.12531, 24965376 PMC4232875

[7] Sylow L Kleinert M Richter EA Jensen TE. Exercise-stimulated glucose uptake—regulation and implications for glycaemic control. Nat Rev Endocrinol. (2017) 13:133–48. doi: 10.1038/nrendo.2016.162, 27739515

[8] Beraki A Magnuson A Särnblad S Åman J Samuelsson U. Increase in physical activity is associated with lower HbA1c levels in children and adolescents with type 1 diabetes: results from a cross-sectional study based on the Swedish pediatric diabetes quality registry (SWEDIABKIDS). Diabetes Res Clin Pract. (2014) 105:119–25. doi: 10.1016/j.diabres.2014.01.029, 24846445

[9] ElSayed NA Aleppo G Aroda VR Bannuru RR Brown FM Bruemmer D . 2. Classification and diagnosis of diabetes: standards of Care in Diabetes-2023. Diabetes Care. (2023) 46:S19–40. doi: 10.2337/dc23-S002, 36507649 PMC9810477

[10] Aronoff SL Berkovitz K Shreiner B Want L. Glucose metabolism and regulation: beyond insulin and glucagon. Diabetes Spectr. (2004) 17:183–90. doi: 10.2337/diaspect.17.3.183

[11] MacMillan F Kirk A Mutrie N Matthews L Robertson K Saunders DH. A systematic review of physical activity and sedentary behavior intervention studies in youth with type 1 diabetes: study characteristics, intervention design, and efficacy. Pediatr Diabetes. (2014) 15:175–89. doi: 10.1111/pedi.12060, 23895512

[12] Cruz Castillo JD Juarez Rojop IE Gonzalez Cortazar M Nolasco Rosales G Gonzalez Castro TB Marin Medina A. Low-carbohydrate diet improves glycemic control in patients with type 1 diabetes: a systematic review. Horizonte Sanit. (2026) 25:e6031. doi: 10.19136/hs.a25.1.6031, 29562676

[13] Soranzo N. Genetic determinants of variability in glycated hemoglobin (HbA1c) in humans: review of recent progress and prospects for use in diabetes care. Curr Diabetes Rep. (2011) 11:562–9. doi: 10.1007/s11892-011-0232-9, 21975967 PMC3207128

[14] Shulman GI. Cellular mechanisms of insulin resistance. J Clin Invest. (2000) 106:171–6. doi: 10.1172/JCI10583, 10903330 PMC314317

[15] Renard E Ikegami H Daher Vianna AG Pozzilli P Brette S Bosnyak Z . The SAGE study: global observational analysis of glycaemic control, hypoglycaemia and diabetes management in T1DM. Diabetes Metab Res Rev. (2021) 37:e3430. doi: 10.1002/dmrr.3430, 33369842 PMC8518876

[16] Pease A Lo C Earnest A Kiriakova V Liew D Zoungas S. Time in range for multiple technologies in type 1 diabetes: a systematic review and network meta-analysis. Diabetes Care. (2020) 43:1967–75. doi: 10.2337/dc19-1785, 32669412

[17] Huerta-Uribe N Ramírez-Vélez R Izquierdo M García-Hermoso A. Association between physical activity, sedentary behavior and physical fitness and glycated hemoglobin in youth with type 1 diabetes: a systematic review and meta-analysis. Sports Med. (2023) 53:111–23. doi: 10.1007/s40279-022-01741-9, 35922715

[18] Parent C Lespagnol E Berthoin S Tagougui S Stuckens C Tonoli C . Continuous moderate and intermittent high-intensity exercise in youth with type 1 diabetes: which protection for dysglycemia? Diabetes Res Clin Pract. (2024) 210:111631. doi: 10.1016/j.diabres.2024.111631, 38513989

[19] Campaigne BN Gilliam TB Spencer ML Lampman RM Schork MA. Effects of a physical activity program on metabolic control and cardiovascular fitness in children with insulin-dependent diabetes mellitus. Diabetes Care. (1984) 7:57–62. doi: 10.2337/diacare.7.1.57, 6705666

[20] Zimmer RT Birnbaumer P Sternad C Zunner BEM Schierbauer J Fritsch M . Impact of a 4-week intensive track and field training intervention on glycaemia in adolescents with type 1 diabetes: the ChilDFiT1 study. Diabetes Obes Metab. (2024) 26:631–41. doi: 10.1111/dom.15352, 37985360

[21] Tomar R Hamdan M Al-Qahtani MH. Effect of low to moderate intensity walking and cycling on glycaemic and metabolic control in type 1 diabetes mellitus adolescent males: a randomized controlled trial. Isokinet Exerc Sci. (2014) 22:237–43. doi: 10.3233/IES-140544

[22] Nazari M Shabani R Hassanzadeh-Rad A Esfandiari MA Dalili S. Effect of concurrent resistance-aerobic training on inflammatory factors and growth hormones in children with type 1 diabetes: a randomized controlled clinical trial. Trials. (2023) 24:519. doi: 10.1186/s13063-023-07553-0, 37568220 PMC10422817

[23] Salem MA AboElAsrar MA Elbarbary NS ElHilaly RA Refaat YM. Is exercise a therapeutic tool for improvement of cardiovascular risk factors in adolescents with type 1 diabetes mellitus? A randomised controlled trial. Diabetol Metab Syndr. (2010) 2:47. doi: 10.1186/1758-5996-2-47, 20618996 PMC3238209

[24] Tunar M Ozen S Goksen D Asar G Bediz CS Darcan S. The effects of pilates on metabolic control and physical performance in adolescents with type 1 diabetes mellitus. J Diabetes Complicat. (2012) 26:348–51. doi: 10.1016/j.jdiacomp.2012.04.006, 22609217

[25] Wong CH Chiang YC Wai JPM Lo FS Yeh CH Chung SC . Effects of a home-based aerobic exercise programme in children with type 1 diabetes mellitus. J Clin Nurs. (2011) 20:681–91. doi: 10.1111/j.1365-2702.2010.03533.x, 21320197

[26] Stratton R Wilson DP Endres RK Goldstein DE. Improved glycemic control after supervised 8-wk exercise program in insulin-dependent diabetic adolescents. Diabetes Care. (1987) 10:589–93. doi: 10.2337/diacare.10.5.589, 3315515

[27] Huttunen NP Lankela SL Knip M Lautala P Kaar ML Laasonen K . Effect of once-a-week training program on physical fitness and metabolic control in children with IDDM. Diabetes Care. (1989) 12:737–40. doi: 10.2337/diacare.12.10.737, 2693013

[28] Maurya S Patil S Singh A Rani I Kaur N Mohanty S . The impact of three months of adjuvant yoga intervention on glycemic control among adolescents with type 1 diabetes: a randomized control trial. Diabetol Metab Syndr. (2025) 17:216. doi: 10.1186/s13098-025-01632-9, 40518537 PMC12168419

[29] Nazari M Minasian V. The impact of organized exercise and non-organized leisured time activity on serum pentraxin3 and C-reactive protein levels, lipid profile and physical fitness in diabetic children. Asian J Sports Med. (2024) 15:e147429. doi: 10.5812/asjsm-147429

[30] Mohammed MHH Al-Qahtani MHH Takken T. Effects of 12 weeks of recreational football (soccer) with caloric control on glycemia and cardiovascular health of adolescent boys with type 1 diabetes. Pediatr Diabetes. (2021) 22:625–37. doi: 10.1111/pedi.13203, 33745203

[31] Petschnig R Wagner T Robubi A Baron R. Effect of strength training on glycemic control and adiponectin in diabetic children. Med Sci Sports Exerc. (2020) 52:2172–2178. doi: 10.1249/MSS.0000000000002356, 32301853

[32] Zhang Y Zhou J Chen X Huang H Fan Z Wang Z. Optimal type and dose of exercise to improve glycated haemoglobin in individuals with type 1 diabetes: a systematic review and Bayesian dose-response network meta-analysis of RCTs. Diabetes Obes Metab. (2025) 27:7146–7157. doi: 10.1111/dom.70115, 40919668

